# New records of *Trachelipusvespertilio* (Budde-Lund, 1896) (Isopoda, Oniscidea) with a description of the male

**DOI:** 10.3897/BDJ.10.e91063

**Published:** 2022-09-29

**Authors:** Miloš Vittori

**Affiliations:** 1 Department of Biology, Biotechnical Faculty, University of Ljubljana, Ljubljana, Slovenia Department of Biology, Biotechnical Faculty, University of Ljubljana Ljubljana Slovenia

**Keywords:** woodlice, terrestrial isopods, Slovenia, crustacean, soil fauna

## Abstract

**Background:**

The existing descriptions of the woodlouse *Trachelipusvespertilio* are based on a single female collected in Croatia in the nineteenth century. No further information on the occurrence of this species has been reported in published literature and the morphology of the male, which may offer additional reliable diagnostic characters, has remained unknown.

**New information:**

On the basis of new material collected in Slovenia, a description of the male morphology of *T.vespertilio* is provided along with a species diagnosis. The rediscovery of this woodlouse after more than a century extends its distribution range to Slovenia.

## Introduction

The genus *Trachelipus* Budde-Lund comprises over 50 recognised species, the species status of some of which is still debated. While some *Trachelipus* species are widely distributed in Europe and fairly well studied, there is very little information available on others, particularly in the Balkans, the centre of diversity of the genus (Schmidt 1997, Schmalfuss 2003, Tomescu et al. 2015, Gongalsky 2017).

The terrestrial isopod *Trachelipusvespertilio* (Budde-Lund, 1896) was originally described as *Porcelliovespertilio* at the end of the nineteenth century (Budde-Lund 1896), but there are no published reports of its being collected or identified since. In his revision of the genus *Trachelipus*, Schmidt recognised this species as valid and provided a more detailed description of Budde-Lund’s material with an illustration of the habitus that depicted the glandular pore fields and the positions of noduli laterales (Schmidt 1997). He examined a female deposited in the British Museum of Natural History (BMNH 1921.10.18.4954), which likely also served as the basis for the original description of the species (Schmidt 1997). The distribution range reported by Budde-Lund, as well as the recorded locality of origin of the female re-examined by Schmidt, is Dalmatia. This broad geographic term does not allow the determination of the type locality. There are no other published reports on the morphology or occurrence of this species. A description of the male may aid in species recognition in the future, since male characters generally show clear interspecific differences and their comparison is often the most straightforward and reliable approach to delimit and identify terrestrial isopod species.

The present work reports on new material of *T.vespertilio* collected or observed in Slovenia and Croatia, representing the first records of the species outside Dalmatia and its first collection in over 120 years. Based on the newly-collected specimens, appendages and the male characters of *T.vespertilio* are described and a diagnosis of the species is provided, facilitating its identification.

## Materials and methods

Isopods were collected by hand in Sežana, Slovenia. Live isopods were imaged using a DigiMicro Profi digital microscope and MicroCapture Pro software (both from DNT). All collected specimens were preserved in 96% ethanol. For light microscopy, pleopods and mouthparts were dissected with a pair of entomological pins and mounted in Euparal (Waldeck). Pereopods, uropods, antennae and mandibles were dissected with tweezers and mounted in glycerol. Whole specimens were imaged in 96% ethanol using an MZ FLIII stereomicroscope, equipped with a DFC425 C digital camera (both from Leica). Dissected appendages were imaged using an AxioImager Z.1 microscope equipped with HRc and MRm digital cameras (all from Zeiss), which was also used for fluorescence imaging with blue excitation (450-490 nm; Zeiss filter set 09) to visualise the structuring of the epicuticle. Drawings were prepared from micrographs using Illustrator software (Adobe). For scanning electron microscopy (SEM), the appendages and cephalothorax were transferred from 96% ethanol to pure acetone, air dried, sputter-coated with platinum and observed using a JSM-7500F field emission SEM (JEOL) at 5 kV acceleration voltage.

All specimens illustrated in the description, as well as three aditional females, were deposited in the Slovenian Museum of Natural History (PMSL-Isopoda-000001–000009). Other specimens are kept in the collection of the author at the University of Ljubljana, Biotechnical Faculty, Department of Biology.

Additional individuals were observed by chance in Paklenica National Park in Croatia, but were not collected. The observed isopods were photographed with a Samsung Galaxy S9 smartphone. To estimate their body-length, they were measured on photographs in comparison to features on the author’s hand.

## Taxon treatments

### Trachelipus vespertilio

(Budde-Lund, 1896)

1926E738-16B8-5DE9-B921-845C64D00DD6

#### Materials

- **Type status:** Other material. **Occurrence:** recordedBy: Miloš Vittori; individualCount: 1; sex: male; lifeStage: adult; **Taxon:** acceptedNameUsage: Trachelipusvespertilio (Budde-Lund, 1896); kingdom: Animalia; phylum: Arthropoda; class: Malacostraca; order: Isopoda; family: Trachelipodidae; genus: Trachelipus; specificEpithet: vespertilio; **Location:** continent: Europe; country: Slovenia; locality: Sežana; verbatimElevation: 369 m; verbatimCoordinates: 45.709194N, 13.862667E; **Identification:** identifiedBy: Miloš Vittori; dateIdentified: 2021; **Event:** eventDate: 19/09/2021; **Record Level:** basisOfRecord: PreservedSpecimen
- **Type status:** Other material. **Occurrence:** recordedBy: Miloš Vittori; individualCount: 2; lifeStage: juvenile; **Taxon:** acceptedNameUsage: *Trachelipusvespertilio* (Budde-Lund, 1896); kingdom: Animalia; phylum: Arthropoda; class: Malacostraca; order: Isopoda; family: Trachelipodidae; genus: Trachelipus; specificEpithet: vespertilio; **Location:** continent: Europe; country: Slovenia; locality: Sežana; verbatimElevation: 369 m; verbatimCoordinates: 45.709194N, 13.862667E; **Identification:** identifiedBy: Miloš Vittori; dateIdentified: 2021; **Event:** eventDate: 03/10/2021; **Record Level:** basisOfRecord: PreservedSpecimen
- **Type status:** Other material. **Occurrence:** recordedBy: Miloš Vittori; individualCount: 2; sex: female; lifeStage: adult; **Taxon:** acceptedNameUsage: *Trachelipusvespertilio* (Budde-Lund, 1896); kingdom: Animalia; phylum: Arthropoda; class: Malacostraca; order: Isopoda; family: Trachelipodidae; genus: Trachelipus; specificEpithet: vespertilio; **Location:** continent: Europe; country: Slovenia; locality: Sežana; verbatimElevation: 369 m; verbatimCoordinates: 45.709194N, 13.862667E; **Identification:** identifiedBy: Miloš Vittori; dateIdentified: 2021; **Event:** eventDate: 30/10/2021; **Record Level:** basisOfRecord: PreservedSpecimen
- **Type status:** Other material. **Occurrence:** recordedBy: Miloš Vittori; individualCount: 1; sex: female; lifeStage: adult; **Taxon:** acceptedNameUsage: *Trachelipusvespertilio* (Budde-Lund, 1896); kingdom: Animalia; phylum: Arthropoda; class: Malacostraca; order: Isopoda; family: Trachelipodidae; genus: Trachelipus; specificEpithet: vespertilio; **Location:** continent: Europe; country: Slovenia; locality: Sežana; verbatimElevation: 369 m; verbatimCoordinates: 45.709194N, 13.862667E; **Identification:** identifiedBy: Miloš Vittori; dateIdentified: 2022; **Event:** eventDate: 26/03/2022; **Record Level:** basisOfRecord: PreservedSpecimen
- **Type status:** Other material. **Occurrence:** recordedBy: Miloš Vittori; individualCount: 3; sex: female; lifeStage: adult; **Taxon:** acceptedNameUsage: *Trachelipusvespertilio* (Budde-Lund, 1896); kingdom: Animalia; phylum: Arthropoda; class: Malacostraca; order: Isopoda; family: Trachelipodidae; genus: Trachelipus; specificEpithet: vespertilio; **Location:** continent: Europe; country: Slovenia; locality: Sežana; verbatimElevation: 369 m; verbatimCoordinates: 45.709194N, 13.862667E; **Identification:** identifiedBy: Miloš Vittori; dateIdentified: 2022; **Event:** eventDate: 02/05/2022; **Record Level:** basisOfRecord: PreservedSpecimen
- **Type status:** Other material. **Occurrence:** recordedBy: Miloš Vittori; individualCount: 4; lifeStage: juvenile; **Taxon:** acceptedNameUsage: *Trachelipusvespertilio* (Budde-Lund, 1896); kingdom: Animalia; phylum: Arthropoda; class: Malacostraca; order: Isopoda; family: Trachelipodidae; genus: Trachelipus; specificEpithet: vespertilio; **Location:** continent: Europe; country: Slovenia; locality: Sežana; verbatimElevation: 369 m; verbatimCoordinates: 45.709194N, 13.862667E; **Identification:** identifiedBy: Miloš Vittori; dateIdentified: 2022; **Event:** eventDate: 11/06/2022; **Record Level:** basisOfRecord: PreservedSpecimen
- **Type status:** Other material. **Occurrence:** recordedBy: Miloš Vittori; individualCount: 4; sex: female; lifeStage: adult; **Taxon:** acceptedNameUsage: *Trachelipusvespertilio* (Budde-Lund, 1896); kingdom: Animalia; phylum: Arthropoda; class: Malacostraca; order: Isopoda; family: Trachelipodidae; genus: Trachelipus; specificEpithet: vespertilio; **Location:** continent: Europe; country: Slovenia; locality: Sežana; verbatimElevation: 369 m; verbatimCoordinates: 45.709194N, 13.862667E; **Identification:** identifiedBy: Miloš Vittori; dateIdentified: 2022; **Event:** eventDate: 06/11/2022; **Record Level:** basisOfRecord: PreservedSpecimen
- **Type status:** Other material. **Occurrence:** recordedBy: Miloš Vittori | Barbara Breznik; individualCount: 1; sex: female; lifeStage: adult; **Taxon:** acceptedNameUsage: *Trachelipusvespertilio* (Budde-Lund, 1896); kingdom: Animalia; phylum: Arthropoda; class: Malacostraca; order: Isopoda; family: Trachelipodidae; genus: Trachelipus; specificEpithet: vespertilio; **Location:** continent: Europe; country: Croatia; locality: Velika Paklenica; verbatimElevation: 40 m; verbatimCoordinates: 44.298374N, 15.461812E; **Identification:** identifiedBy: Miloš Vittori; dateIdentified: 2022; **Event:** eventDate: 14/06/2022; **Record Level:** basisOfRecord: LivingSpecimen
- **Type status:** Other material. **Occurrence:** recordedBy: Miloš Vittori | Barbara Breznik; individualCount: 1; sex: male; lifeStage: adult; **Taxon:** acceptedNameUsage: *Trachelipusvespertilio* (Budde-Lund, 1896); kingdom: Animalia; phylum: Arthropoda; class: Malacostraca; order: Isopoda; family: Trachelipodidae; genus: Trachelipus; specificEpithet: vespertilio; **Location:** continent: Europe; country: Croatia; locality: Velika Paklenica; verbatimElevation: 387 m; verbatimCoordinates: 44.334795N, 15.476142E; **Identification:** identifiedBy: Miloš Vittori; dateIdentified: 2022; **Event:** eventDate: 15/06/2022; **Record Level:** basisOfRecord: LivingSpecimen

#### Description

**Figure 1.**
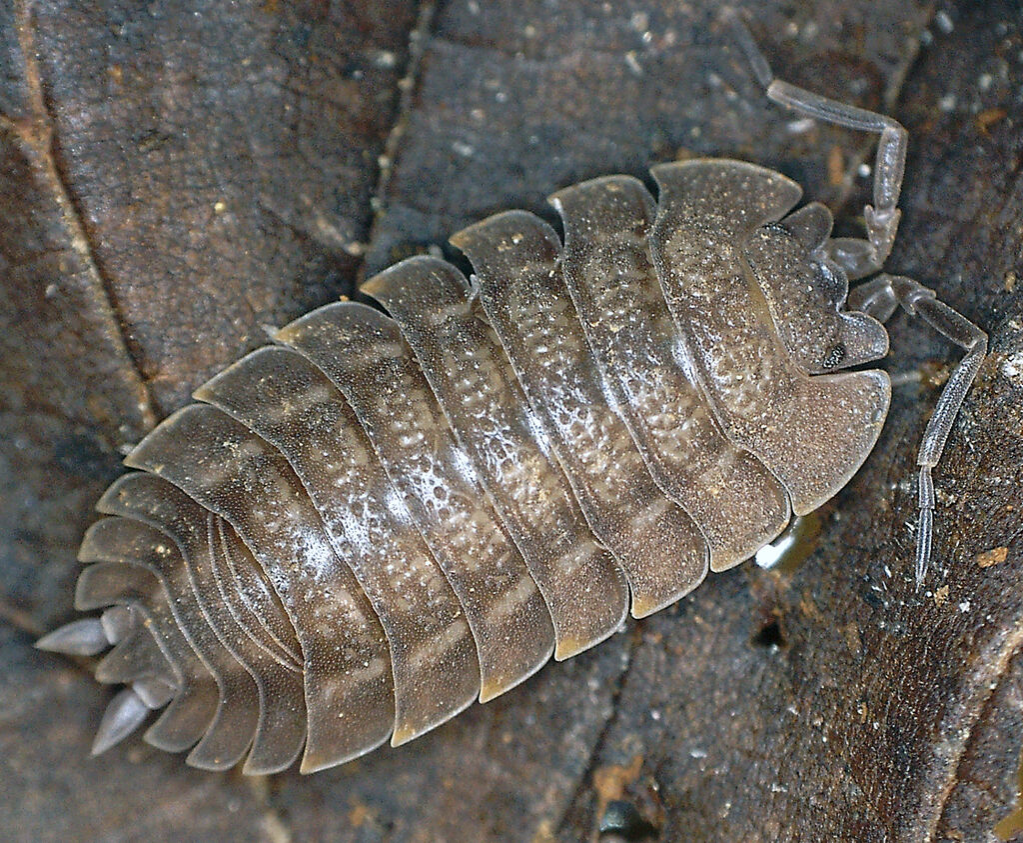
Live female *Trachelipusvespertilio* (11 mm), collected in Sežana, Slovenia.

**Figure 2.**
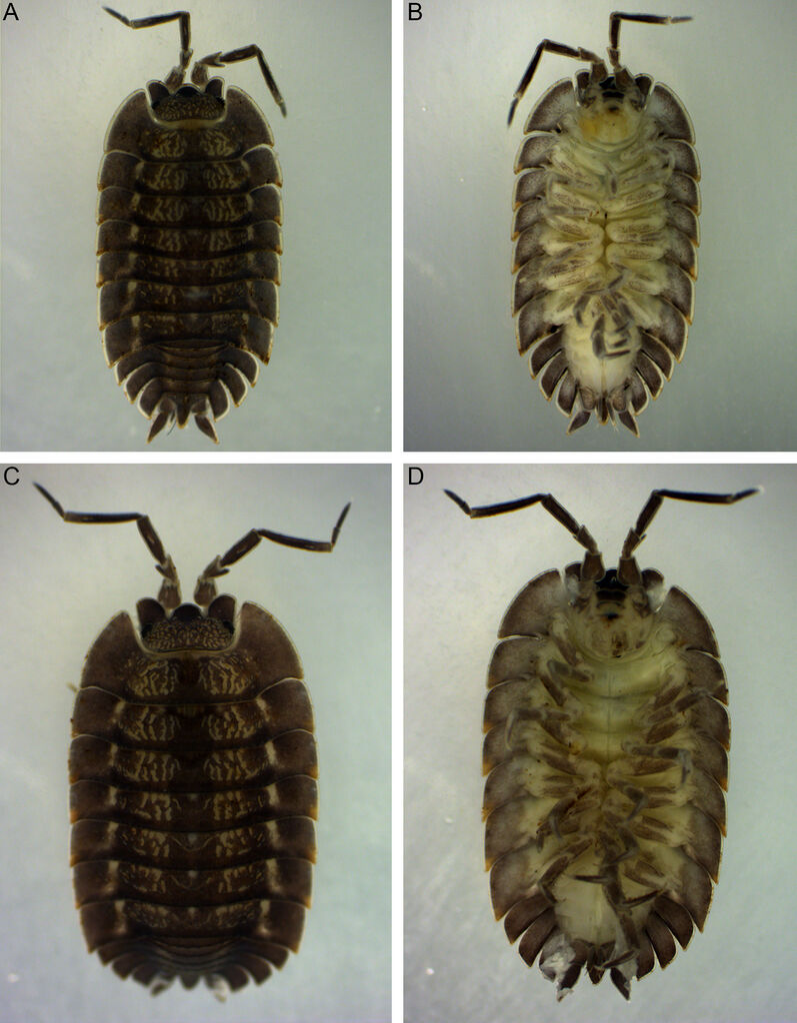
*Trachelipusvespertilio*, habitus of specimens collected in Sežana, Slovenia. **A** male, 11 mm, dorsal view; **B** male, ventral view; **C** female, 12 mm, dorsal view; **D** female, ventral view.

Colour dorsally light greyish-brown with pale patches on bases of epimera on pereonites 2-7 and a median row of small pale patches on anterior edges of pereon tergites 4-7. Pleon with two faint lighter longitudinal lines. Ventrally, epimera and pereopods light grey, sternites and pleopods white. Posterior corners of pereon epimera in most specimens with faint orange patches less than one-fifth the length of epimeron (Figs 1, 2).

**Figure 3.**
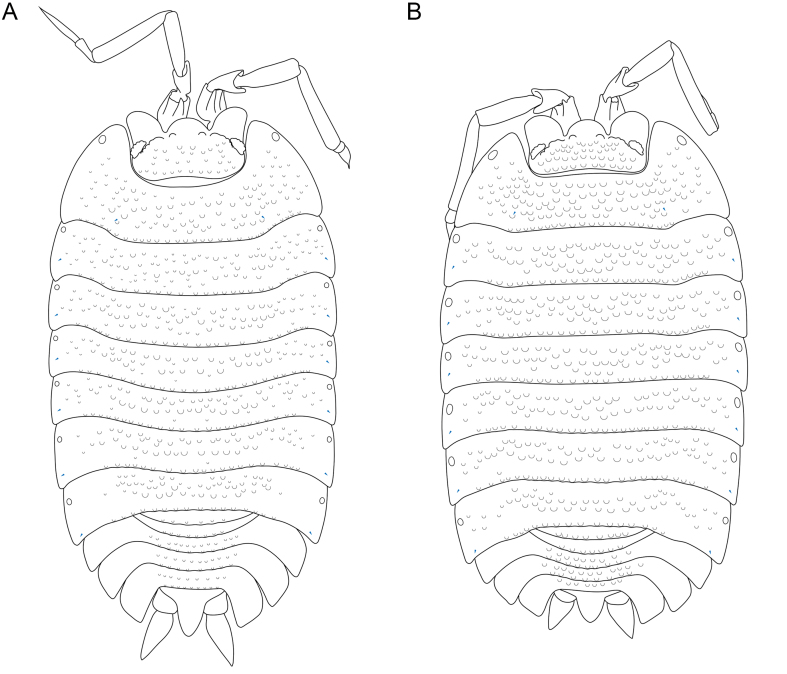
*Trachelipusvespertilio*, habitus. **A** male, 11 mm, collected in Sežana, Slovenia; **B** female, 12 mm, collected in Sežana, Slovenia. Noduli laterales are depicted in blue.

**Figure 4.**
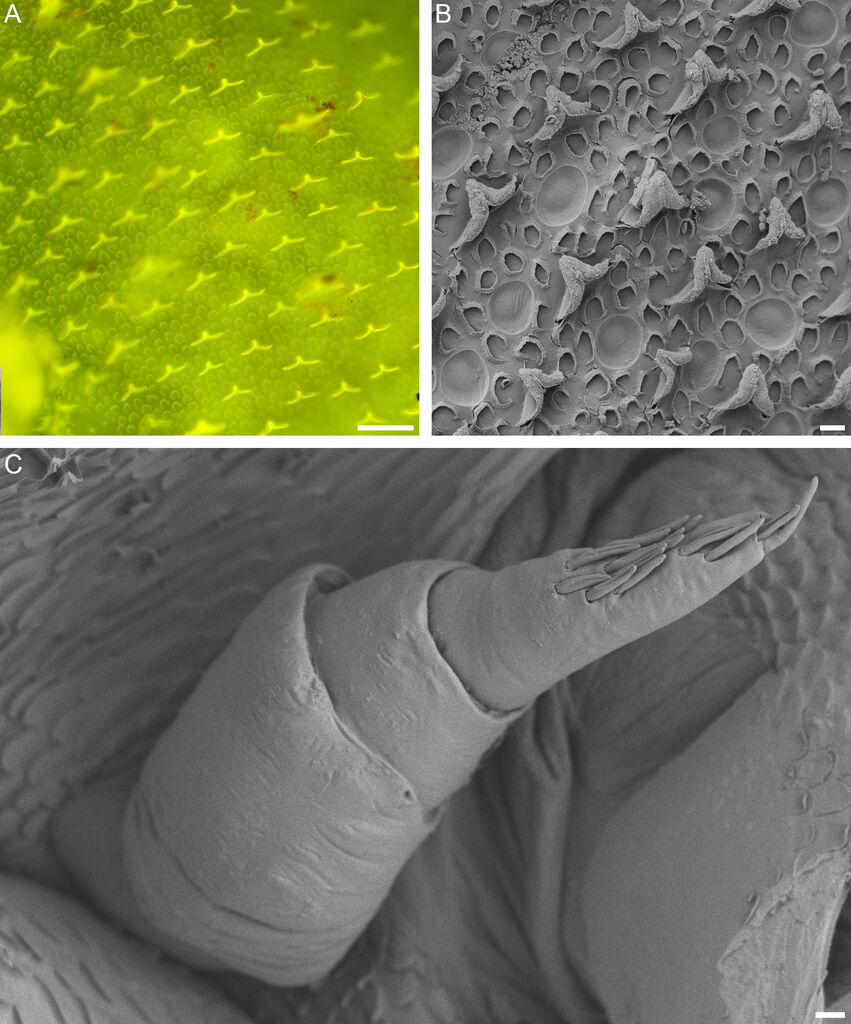
Structure of the dorsal body surface and antennula of *Trachelipusvespertilio*. **A** fluorescence micrograph of the epicuticular surface showing strongly fluorescent tricorns and scales; **B** scanning electron micrograph of tricorns, scales and circular depressions on dorsal body surface; **C** antennula of *Trachelipusvespertilio* male, 11 mm, collected in Sežana, Slovenia. Scale bars: 50 µm (**A**), 10 µm (**B** and **C**).

Cephalothorax and pereon tergites strongly tuberculate (Fig. 3). Tuberculation more pronounced on pereonites 1-4 than on peronites 5-7, pereon epimera and pleon weakly tuberculate. Row of prominent tubercules present on posterior margins of tergites; as a result, posterior margins of pereon tergites 5-7 and pleon tergites slightly wavy, but not serrate. Dorsal body surface covered with tricorns wider than their length and with semilunar and annular scales (Fig. 4A and B).

**Figure 5.**
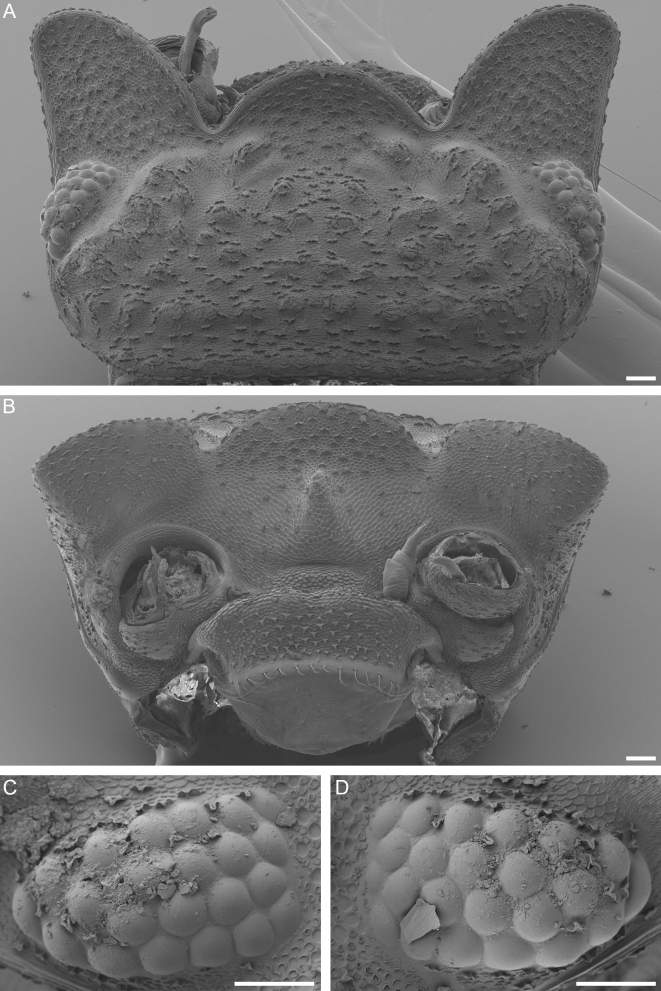
*Trachelipusvespertilio*, cephalothorax of male, 11 mm, collected in Sežana, Slovenia. **A** dorsal view of cephalothorax; **B** frontal view of cephalothorax; **C** right eye; **D** left eye. Scale bars: 100 µm.

Lateral cephalic lobes twice as long as eyes, with straight outer margins and curved inner margins (Figs 1, 2, 3, 5). Median cephalic lobe half as long as lateral lobes and evenly rounded. Angles between cephalic lobes acute. Distal margins of all lobes curved upwards. Pair of prominent tubercles at base of median lobe near its lateral edges.

Eyes (Fig. 5C and D) composed of 22-25 ommatidia. Eyes less than half as long as cephalothorax, approximately equal to length of median cephalic lobe.

Posterior margins of pereon epimera concave. Concave edges on pereonite 1 not evenly curved, instead with straight mid-section (Figs 1, 2, 3).

Glandular pore fields on pereon epimera nearly circular, with diameter larger than distance to lateral margin of epimera (Fig. 3).

Noduli laterales (Fig. 3) on pereonite 1 approximately equidistant from mid-line as from lateral margin of pereonite, on other pereonites posterior to glandular pore fields and aligned with their median margins. Noduli laterales on pereonites 2-5 closer to lateral than posterior margin of each pereonite; on pereonite 6, their distance from posterior and lateral margin approximately equal; on pereonite 7, more than twice as far from lateral than posterior margin.

Pleotelson slightly longer than wide, with concave lateral margins converging strongly in anterior half of pleotelson and gradually in posterior half. Apex of pleotelson rounded (Fig. 3).

Antennula (Fig. 4C) consisting of 3 articles. Second article half as long as basal article and terminal article approximately as long as basal article. Terminal article bearing 15 aesthetascs in male examined.

**Figure 6.**
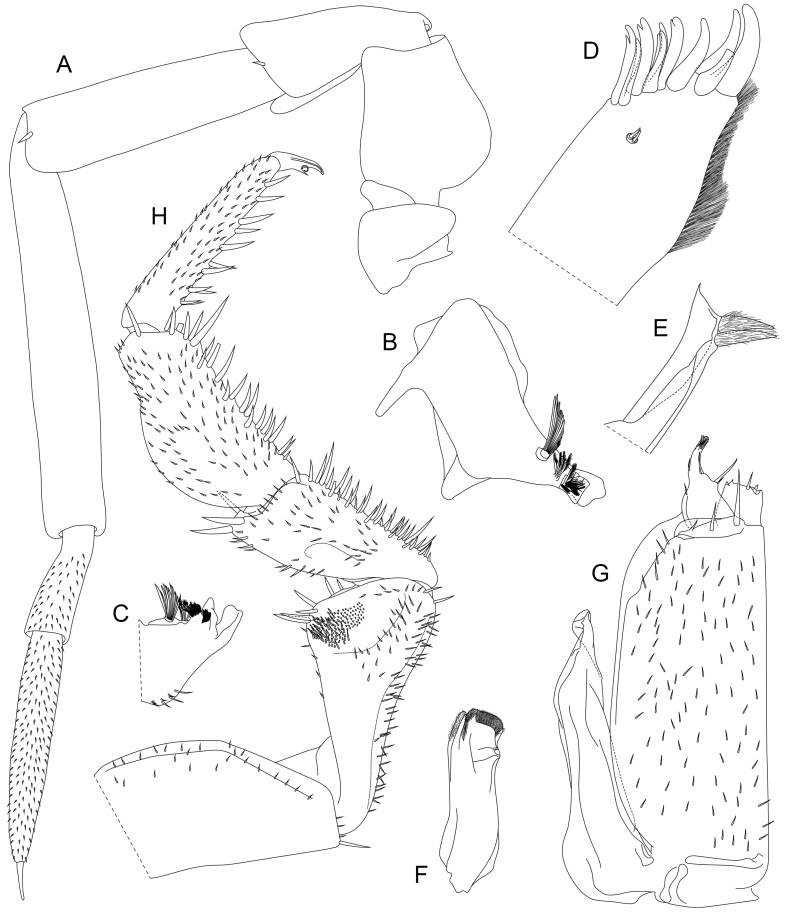
Appendages of cephalothorax and pereon,*Trachelipusvespertilio*. **A** antenna, ventral view; **B** left mandible; **C** right mandible; **D** outer branch of maxilllula; **E** inner branch of maxillula; **F** maxilla; **G** maxilliped; **H** pereopod 7, anterior view. **A**–**D** and **F**–**H**: male, 11 mm, collected in Sežana, Slovenia **E**: female, 11 mm, collected in Sežana, Slovenia.

Antenna (Fig. 6A) with article 2 medially rounded, its dorsal surface with ridge ending distally in a pointed projection. Article 3 dorsally with distal projection with rounded tip. Flagellum composed of two articles, distal article at least twice as long as proximal article.

Mandibles (Fig. 6B and C) characteristic of genus (Schmidt 1997). Lacinia mobilis with two teeth, proximally to it hairy lobe with two penicils; proximally to hairy lobe row of 4 penicils in male examined. Molar penicil with tuft of more than 10 composite setae arising from common tubercle.

Maxillula (Fig. 6D and E) outer branch with 10 teeth, 6 teeth in median group, all except tooth 2 and 5 with split tips, lateral group of 4 teeth with simple tips; two small and stout subapical setae on caudal surface close together, in contact. Apex of inner branch medially with two penicils.

Maxilla (Fig. 6F) characteristic of genus (Schmidt 1997), bilobed, both lobes covered with thin setules. Subapical tubercle as elongate ridge perpendicular to long axis of maxilla.

Maxilliped (Fig. 6G) characteristic of genus (Schmidt 1997). Basal article of palp with 2 large setae, second article with one large and one smaller seta on median margin, distal margin with single large seta.

**Figure 7.**
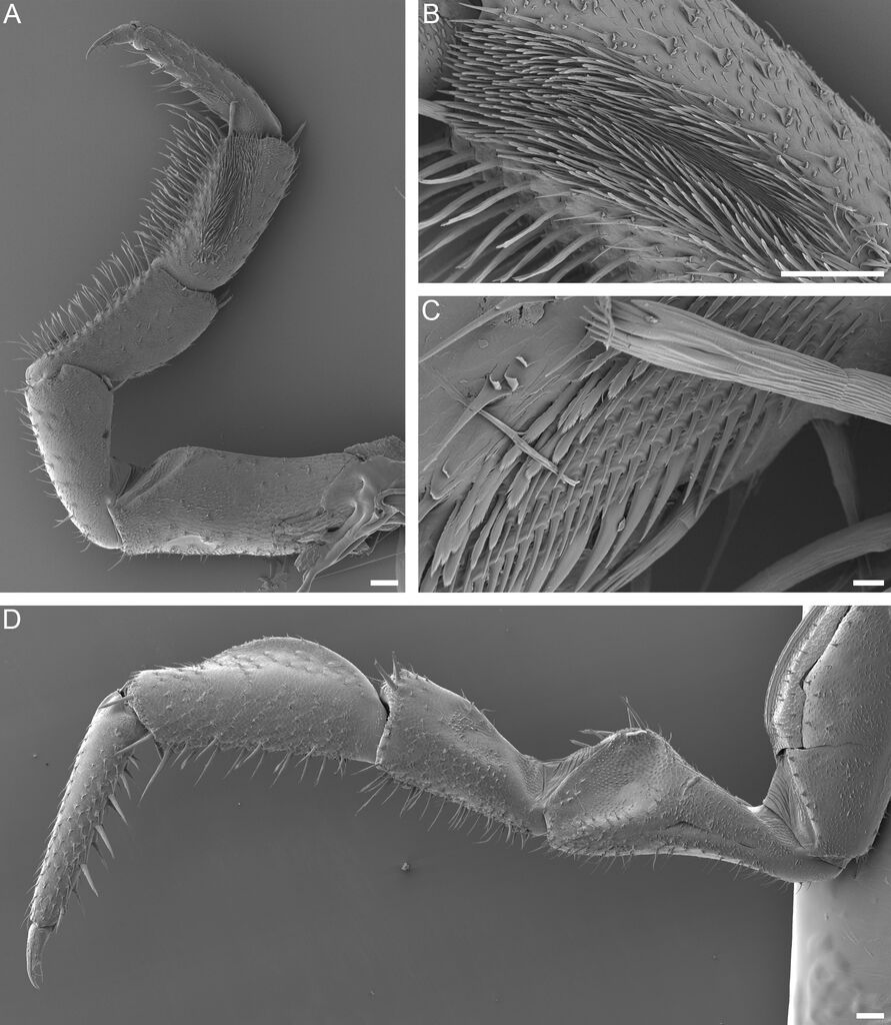
*Trachelipusvespertilio*, pereopods 1 and 7 of male, 11 mm, collected in Sežana, Slovenia. **A** pereopod 1, anterior view; **B** patch of setae on merus of pereopod 1; **C** patch of setae on propodus of pereopod 1; **D** pereopod 7, anterior view. Scale bars: 100 µm (**A**, **B** and **D**), 10 µm (**C**).

Male pereopod 1 (Fig. 7A-C) with elongate, dense patch of setules on frontal side of merus. Frontal side of propodus in its proximal half with elongate patch of cuspidate setae arranged in several rows.

Male pereopod 7 (Fig. 6H and Fig. 7D) with ventrally concave ischium, frontal side of ischium bearing semi-circular pit covered with setules increasing in length towards dorsal margin of pit. Pit delimited ventrally by slightly concave ridge. Carpus dorsally with evenly curved crest reaching three-quarters of carpus length, crest highest at mid-point.

**Figure 8.**
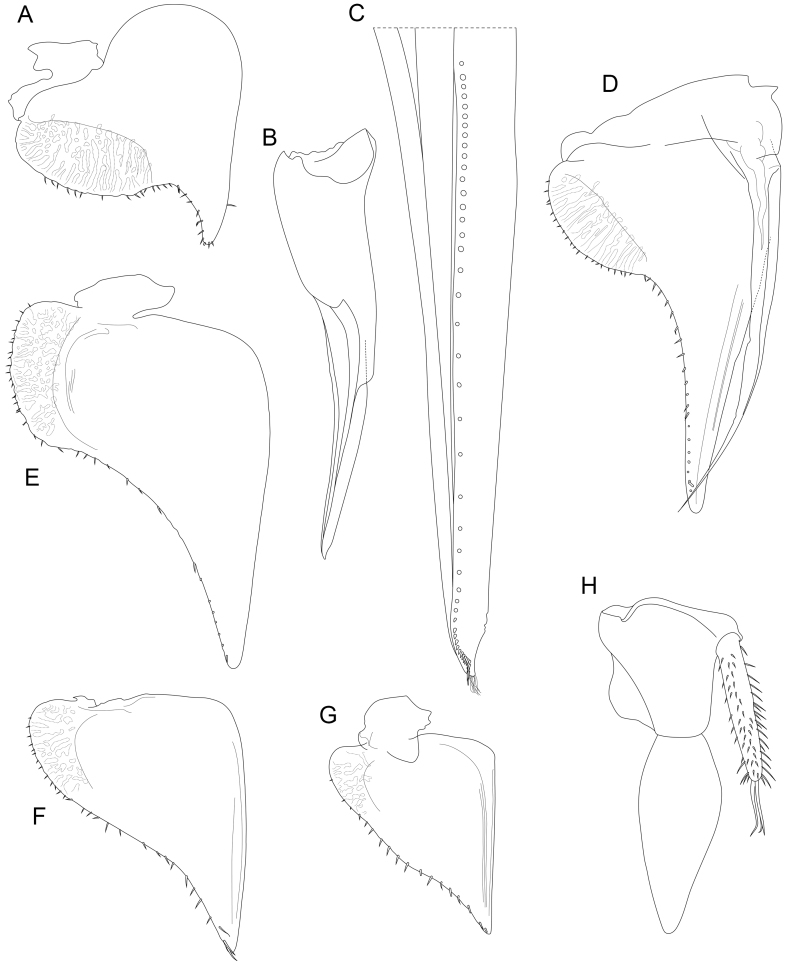
Appendages of pleon, *Trachelipusvespertilio* male, 11 mm, collected in Sežana, Slovenia **A** pleopod 1, exopod; **B** pleopod 1, endopod; **C** pleopod 1, endopod at high magnification; **D** pleopod 2; **E** pleopod 3; **F** pleopod 4; **G** pleopod 5; **H** uropod.

Pleopod exopodites 1-5 (Fig. 8A-G) with lungs characteristic of genus (Schmidt 1997). Lateral margins of lung fields on all pleopods convex, rounded, on pleopods 1-4 with numerous (more than 10) setae, on pleopod 5 with fewer than 5 setae.

Male pleopod 1 exopod (Fig. 8A) with stout, evenly curved apical projection, its length equal to width of lung field. Pleopod 1 endopod (Fig. 8B and C) with row of stout setae and apical tuft of setules. Row of setae curved near apex of endopod and straight at its very tip (Fig. 8C).

Male pleopod 2 exopod (Fig. 8D) twice as long as wide, its tip slightly curved inwards. Pleopod 2 endopod (Fig. 8D) as long as exopod, slender, curving outwards.

Uropod (Fig. 8H) protopod approximately as long as wide, exopod 1.5 times longer than protopod and 2.5 times longer than wide, widest at one-third its length, with slightly convex lateral margin and strongly convex median margin. Uropod endopod with parallel sides, setose, approximately as long as protopod.

#### Diagnosis

Lateral lobes of cephalothorax more than twice as long as median lobe and eyes. Acute angles between cephalic lobes. Distal article of flagellum of antenna at least twice as long as proximal article. Concave posterior margins of epimera on pereonite 1 not evenly curved, instead with straight segment in middle. Glandular pore fields on all pereonites circular and closer than their diameter to lateral edges of epimera. Carpus of male pereopod 7 dorsally with symmetrical, evenly curved crest reaching two-thirds length of carpus, highest point of crest in the middle. Lung fields on all pleopod exopods with convex, rounded edges. Male pleopod 1 exopodite with stout uniformly curved apical projection as long as width of lung field. Male pleopod 1 endopod with row of setae that curves as it approaches endopod apex and straightens abruptly at apex. Apex with tuft of setules.

#### Distribution

East coast of the Adriatic Sea between Velebit (Croatia) and the Karst Plateau (Slovenia).

#### Notes

Amongst the material from Sežana, six juveniles were collected: two in October 2021 and an additional four in June 2022. The cephalic lobes are smaller in juveniles and the difference in length between the median and lateral lobes is not as pronounced as in adults. Three ovigerous females were collected at the same locality in June 2022.

**Figure 9.**
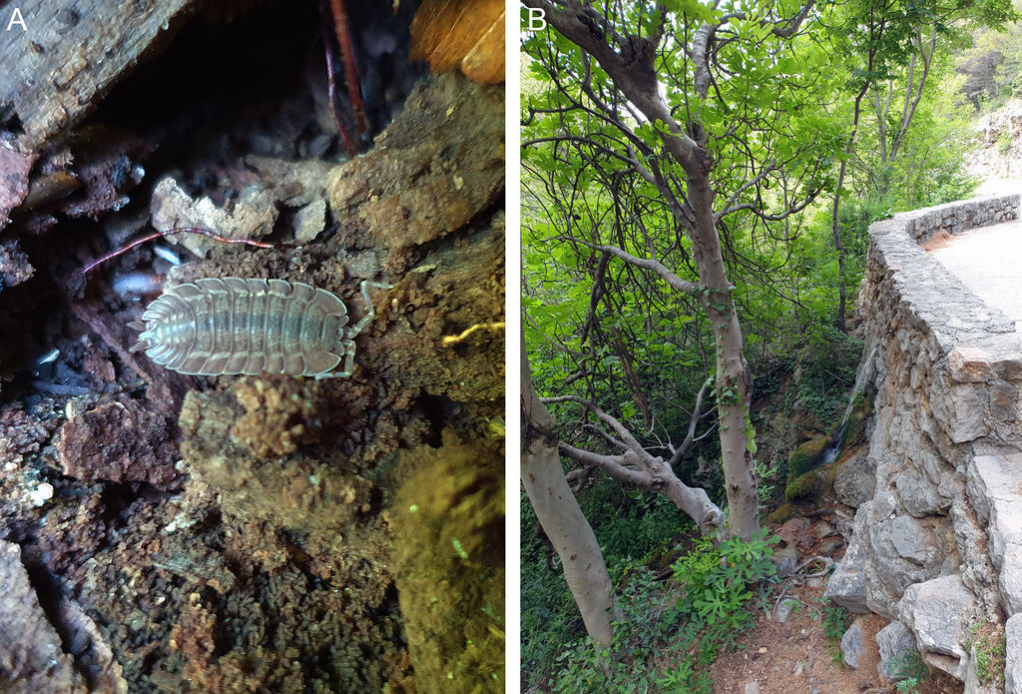
*Trachelipusvespertilio* observed in Paklenica, Croatia. **A** photograph of male, spotted above Lugarnica cottage in Velika Paklenica Canyon; **B** spring on the side of the road in Velika Paklenica Canyon where a female was found.

Two additional individuals, a male (Fig. 9A) and a female, were observed in Velika Paklenica Canyon in Paklenica National Park in Croatia. These isopods were found in a deciduous forest in very humid places, the female under a stone near a spring (Fig. 9B) and the male under moist decaying wood.

## Discussion

The examined specimens of *T.vespertilio* were collected in Sežana in the coastal area of Slovenia. The isopods were found under wooden boards and large stones in green areas between multi-storey residential buildings. The presence of juveniles and ovigerous females demonstrates that the isopods in Sežana reproduce, suggesting that they are permanent residents of the region, although found only in a synanthropic habitat so far. Our observations from Croatia indicate that this species also lives in forests and its choice of habitat may depend on the local climate. The isopods collected in Sežana were slightly smaller than the specimen previously described (Budde-Lund 1896, Schmidt 1997), as their body length did not exceed 12 mm. By contrast, individuals observed in Croatia were larger than the specimens from Slovenia, approximately 14 mm in length and comparable with the reported size of Budde-Lund’s specimen.

The lack of records of *T.vespertilio* after its original discovery may be due to the species being overlooked for a time. Prior to the revision of the genus *Trachelipus* (Schmidt 1997), there were no illustrations of *T.vespertilio* and only the original description could be used for its identification. In addition, *T.vespertilio* was not included in most identification keys, including the influential key by Schmölzer (Schmölzer 1965). As a result, it was difficult to identify it if it had been collected. Nevertheless, the region of Slovenia where *T.vespertilio* is present was well studied less than twenty years ago without finding the species (Vilisics and Lapanje 2005). It is, therefore, likely that *T.vespertilio* is expanding its range northwards in synanthropic habitats and has only recently established populations in Slovenia. Although new records expand the distribution range of *T.vespertilio*, it remains small, making the species vulnerable to extinction.

As discussed by Schmidt, male characters important to species identification in the genus *Trachelipus* include the shape of male pereopod 7, especially the carpus and the shape of male pleopod 1 (Schmidt 1997). Other taxonomically significant characters in this genus include the size and position of the glandular pore fields on the epimera, the structure of pleopodal lungs, the size and shape of the cephalic lobes and granulation of the tergites. In his revision of the genus, Schmidt considered *T.vespertilio* a valid species, based on the shape and relative size of the cephalic lobes, with the lateral lobes “far surpassing the median lobe in size and about twice as long as eyes”. This certainly describes the species well; although its cephalic lobes are not remarkably large, the eyes of *T.vespertilio* are relatively smaller than in other *Trachelipus* species with well-developed cephalic lobes. This, in combination with other characters, such as the convex edges of lung fields with numerous setae, the positions of glandular pore fields near the edges of pereon epimera, the straight edge of the concavities on the posterior edge of pereonite 1, makes *T.vespertilio* easily distinguishable from other *Trachelipus* species inhabiting the region.

Some other European species of *Trachelipus*, such as *Trachelipuscamerani* (Tua, 1900) and *Trachelipusrhinoceros* (Budde-Lund, 1885) may have similarly-shaped cephalic lobes as *T.vespertilio* and overlapping distribution ranges. The median cephalic lobe usually has a straight front edge in *T.camerani*, differentiating it from *T.vespertilio*, but it is reportedly highly variable in *T.rhinoceros* (Schmidt 1997). Nevertheless, these species can be easily distinguished from *T.vespertilio*. In both *T.camerani* and *T.rhinoceros*, the posterior edges of the tergites are serrated. Furthermore, the distal article of the antennal flagellum is less than twice as long as the proximal article in these two species. Other differences are apparent in male characters, such as the shape of pereopod 7 and the position of the tuft of setules on the endopod of pleopod 1, which is subapical in *T.camerani* and *T.rhinoceros*.

Another morphologically similar species is *Trachelipusratzeburgii* (Brandt, 1833), which resembles *T.vespertilio* in characters such as the position of glandular pore fields, the relative position of noduli laterales, the appearance of the cephalic lobes and the shape of the antenna. An important difference between *T.vespertilio* and *T.ratzeburgii* is the relative size of the eyes, which are longer than half the length of both the cephalothorax the lateral cephalic lobes in *T.ratzeburgii*. Furthermore, the margins of lung fields on most pleopods are not convex in *T.ratzeburgii* and the tuberculation of the tergites is less pronounced. The two species also differ in colouration, although this is likely to vary to some extent. Further differences are in the crest on the carpus of male pereopod 7, which is highest proximally in *T.ratzeburgii* and highest in the middle in *T.vespertilio*, but this feature is also expected to vary, at least in *T.ratzeburgii* (Tomescu et al. 2015). Other male characters are similar in the two species.

Although a total of 17 specimens were collected in this study, only one male was unfortunately available for microscopic examination. This makes it impossible to assess the possible variability of male characters between individuals of *T.vespertilio*, which may be considerable in some species of the genus (Tomescu et al. 2015).

## Supplementary Material

XML Treatment for Trachelipus vespertilio
